# The genome sequence of the Asparagus Beetle, *Crioceris asparagi *(Linnaeus, 1758)

**DOI:** 10.12688/wellcomeopenres.23460.1

**Published:** 2025-01-15

**Authors:** Michael F. Geiser, Freya Read, Maxwell V.L. Barclay, Natural History Museum Genome Acquisition Lab, Darwin Tree of Life Barcoding collective, Wellcome Sanger Institute Tree of Life Management, Samples and Laboratory team, Wellcome Sanger Institute Scientific Operations: Sequencing Operations, Wellcome Sanger Institute Tree of Life Core Informatics team, Tree of Life Core Informatics collective, Darwin Tree of Life Consortium

**Affiliations:** 1 Natural History Museum, London, England, UK; 2 People's Trust for Endangered Species, London, England, UK

**Keywords:** Crioceris asparagi, Asparagus Beetle, genome sequence, chromosomal, Coleoptera

## Abstract

We present a genome assembly from an individual male specimen of *Crioceris asparagi* (Asparagus Beetle; Arthropoda; Insecta; Coleoptera; Chrysomelidae). The genome sequence has a total length of 639.30 megabases. Most of the assembly (93.04%) is scaffolded into 9 chromosomal pseudomolecules, including the X and Y sex chromosomes. The mitochondrial genome has also been assembled and is 15.76 kilobases in length. Gene annotation of this assembly on Ensembl identified 26,673 protein-coding genes.

## Species taxonomy

Eukaryota; Opisthokonta; Metazoa; Eumetazoa; Bilateria; Protostomia; Ecdysozoa; Panarthropoda; Arthropoda; Mandibulata; Pancrustacea; Hexapoda; Insecta; Dicondylia; Pterygota; Neoptera; Endopterygota; Coleoptera; Polyphaga; Cucujiformia; Chrysomeloidea; Chrysomelidae; Criocerinae; *Crioceris*; *Crioceris asparagi* (Linnaeus, 1758) (NCBI:txid131627)

## Background

*Crioceris asparagi* (Linnaeus, 1758), commonly known as the “Asparagus Beetle” is one of about 250 species of the leaf beetle family (Chrysomelidae) found on the British Isles ( Duff, 2018). Chrysomelidae are one of the largest families of Coleoptera, with over 40,000 currently valid species. *Crioceris* Geoffroy, 1762 is the type genus of the subfamily Criocerinae, represented by seven species in Britain ( Duff, 2018) and about 1500 worldwide ( Monrós, 1960; Vencl & Leschen, 2014). *Crioceris* is native to the Palaearctic, Oriental and Afrotropical regions, counting about 30 described species ( Bezděk & Sekerka, 2024; Heinze, 1962; Kimoto & Gressitt, 1979; Monrós, 1960). *C. asparagi* is currently the only *Crioceris* on the British checklist, though occasional records of two more species, *C. duodecimpunctata* (Linnaeus, 1758) and *C. macilenta* Weise, 1881, exist. The latter two are considered non-established accidental introductions ( Duff, 2018).

Adults and larvae of *C. asparagi* feed externally and conspicuously on the leaves and stems of wild, and more often cultivated, asparagus ( *Asparagus* spp.: Asparagaceae) and can be a pest ( Rheinheimer & Hassler, 2018). Pupation takes place in the soil at the base of the plant. Immature stages are described by Steinhausen ( 1994; 2001).

The native distribution of *C. asparagi* includes almost all of Europe except the far North, the Middle East, Central Asia and Siberia ( Bezděk & Sekerka, 2024). It has been an important non-native pest of asparagus in the USA and Canada for more than a century ( Chittenden, 1917).

Cox (2007) shows it to be widespread in England, but with only scarce records towards the North and West. He mentions a few scattered records in Wales, but considers it absent from Scotland and Northern Ireland. He also observes that its British distribution much more closely matches the range of cultivated asparagus, than the more coastal distribution of the uncommon wild asparagus. NBN Atlas Partnership (2024) shows that *C. asparagi* has apparently increased both its range and abundance, now also occurring in a few places in Scotland, but remains very scarce in Wales. Whether it is indigenous to the UK is still a matter of speculation, but Fowler (1890) believed it to be, so if it is an introduction, it is a long-standing one.

*C. asparagi* cannot easily be confused with any other British leaf beetle. Its cylindrical shape is somewhat atypical for Chrysomelidae, but not unusual for a Criocerinae. The elegant blue metallic or shiny black ladder-like elytral pattern on top of a whitish background, with reddish elytral margins and pronotum is very much unique. Variations with reduced or expanded elytral pattern exist ( Warchałowski, 2010), but those individuals are still easily recognisable among other Criocerinae. Specimens with a dark spot in the middle of the red pronotum seem to be unique to the eastern Mediterranean and Middle East and are sometimes classified as subspecies *maculipes* (Germar, 1834). Specimens with more coarsely punctate pronotum from Central Asia have previously been assigned to another subspecies, *turkestanica* Medvedev, 1955 (a synonym according to Bezděk & Sekerka, 2024). In the British fauna, only the nominate subspecies has been recorded ( Duff, 2016; Warchałowski, 2010).

**Figure 1.**
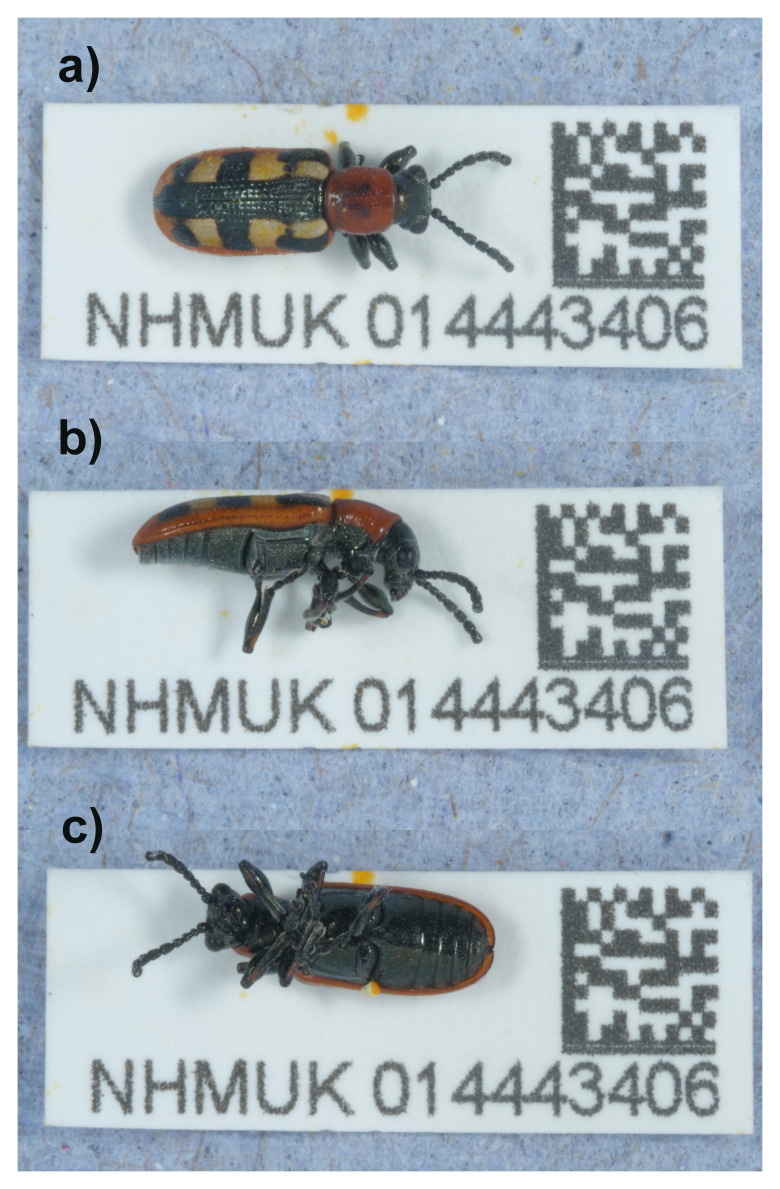
Photographs of the *Crioceris asparagi* (icCriAspa1) specimen used for genome sequencing.

The genome of the Asparagus Beetle, *Crioceris asparagi*, was sequenced as part of the Darwin Tree of Life Project, a collaborative effort to sequence all named eukaryotic species in the Atlantic Archipelago of Britain and Ireland. Here we present a chromosomally complete genome sequence for *Crioceris asparagi*, based on a male specimen collected from cultivated asparagus at an allotment at Winsford Gardens, Penge, England, United Kingdom ( Figure 1). This appears to be the first genome for a member of the subfamily Criocerinae to be made available.

## Genome sequence report

The genome of *Crioceris asparagi* ( Figure 1) was sequenced using Pacific Biosciences single-molecule HiFi long reads, generating a total of 25.24 Gb (gigabases) from 2.11 million reads, providing an estimated 36-fold coverage. Primary assembly contigs were scaffolded with chromosome conformation Hi-C data, which produced 164.92 Gb from 1,092.17 million reads. Specimen and sequencing details are summarised in Table 1.

**Table 1.** Specimen and sequencing data for *Crioceris asparagi*.

Project information			
**Study title**	Crioceris asparagi (common asparagus beetle)		
**Umbrella BioProject**	PRJEB59077		
**Species**	*Crioceris asparagi*		
**BioSample**	SAMEA111458050		
**NCBI taxonomy ID**	131627		
Specimen information			
**Technology**	**ToLID**	**BioSample** **accession**	**Organism part**
**PacBio long read sequencing**	icCriAspa1	SAMEA111458352	abdomen
**Hi-C sequencing**	icCriAspa1	SAMEA111458147	Head and thorax
Sequencing information			
**Platform**	**Run accession**	**Read count**	**Base count (Gb)**
**Hi-C Illumina NovaSeq 6000**	ERR10802453	1.09e+09	164.92
**PacBio Sequel IIe**	ERR10802387	2.11e+06	25.24

Assembly errors were corrected by manual curation, including 12 missing joins or mis-joins. This reduced the scaffold number by 1.25% and increased the scaffold N50 by 106.78%. The final assembly has a total length of 639.30 Mb in 709 sequence scaffolds, with 161 gaps, and a scaffold N50 of 90.6 Mb ( Table 2).

**Table 2.** Genome assembly data for *Crioceris asparagi*, icCriAspa1.1.

Genome assembly		
Assembly name	icCriAspa1.1	
Assembly accession	GCA_958507055.1	
*Accession of alternate haplotype*	*GCA_958501965.1*	
Span (Mb)	639.30	
Number of contigs	871	
Number of scaffolds	709	
Longest scaffold (Mb)	131.42	
Assembly metrics *		*Benchmark*
Contig N50 length (Mb)	5.5	*≥ 1 Mb*
Scaffold N50 length (Mb)	90.6	*= chromosome N50*
Consensus quality (QV)	58.8	*≥ 40*
*k*-mer completeness	primary: 90.19%; alternate: 61.69%; combined: 98.64%	*≥ 95%*
BUSCO **	C:98.4%[S:97.6%,D:0.8%], F:0.2%,M:1.4%,n:2,124	*S > 90%*, *D < 5%*
Percentage of assembly mapped to chromosomes	93.04%	*≥ 90%*
Sex chromosomes	XY	*localised homologous pairs*
Organelles	Mitochondrial genome: 15.76 kb	*complete single alleles*
Genome annotation of assembly GCA_958507055.1 at Ensembl		
Number of protein-coding genes	26,673	
Number of gene transcripts	26,898	

* Assembly metric benchmarks are adapted from Rhie *et al.* (2021) and the Earth BioGenome Project Report on Assembly Standards September 2024. ** BUSCO scores based on the endopterygota_odb10 BUSCO set using version 5.3.2. C = complete [S = single copy, D = duplicated], F = fragmented, M = missing, n = number of orthologues in comparison. A full set of BUSCO scores is available at https://blobtoolkit.genomehubs.org/view/icCriAspa1_1/dataset/icCriAspa1_1/busco.

**Figure 2.**
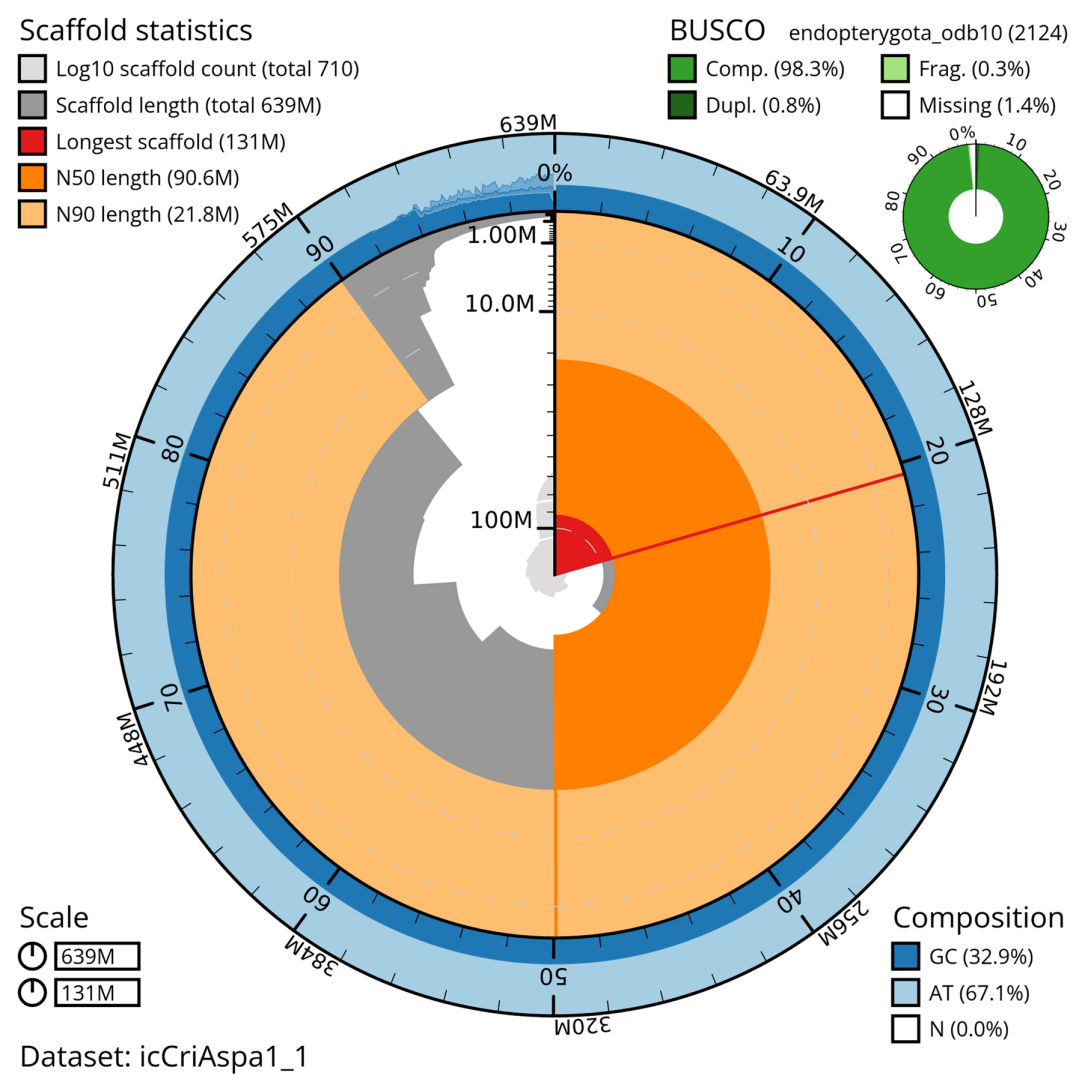
Genome assembly of *Crioceris asparagi*, icCriAspa1.1: metrics. The BlobToolKit snail plot provides an overview of assembly metrics and BUSCO gene completeness. The circumference represents the length of the whole genome sequence, and the main plot is divided into 1,000 equal-sized bins around the circumference. The outermost blue tracks display the distribution of GC, AT, and N percentages across the bins. Scaffolds are arranged clockwise from longest to shortest and are depicted in dark grey. The longest scaffold is indicated by the red arc, and the deeper orange and pale orange arcs represent the N50 and N90 lengths. A light grey spiral at the centre shows the cumulative scaffold count on a logarithmic scale. A summary of complete, fragmented, duplicated, and missing BUSCO genes in the endopterygota_odb10 set is presented at the top right. An interactive version of this figure is available at https://blobtoolkit.genomehubs.org/view/icCriAspa1_1/dataset/icCriAspa1_1/snail.

**Figure 3.**
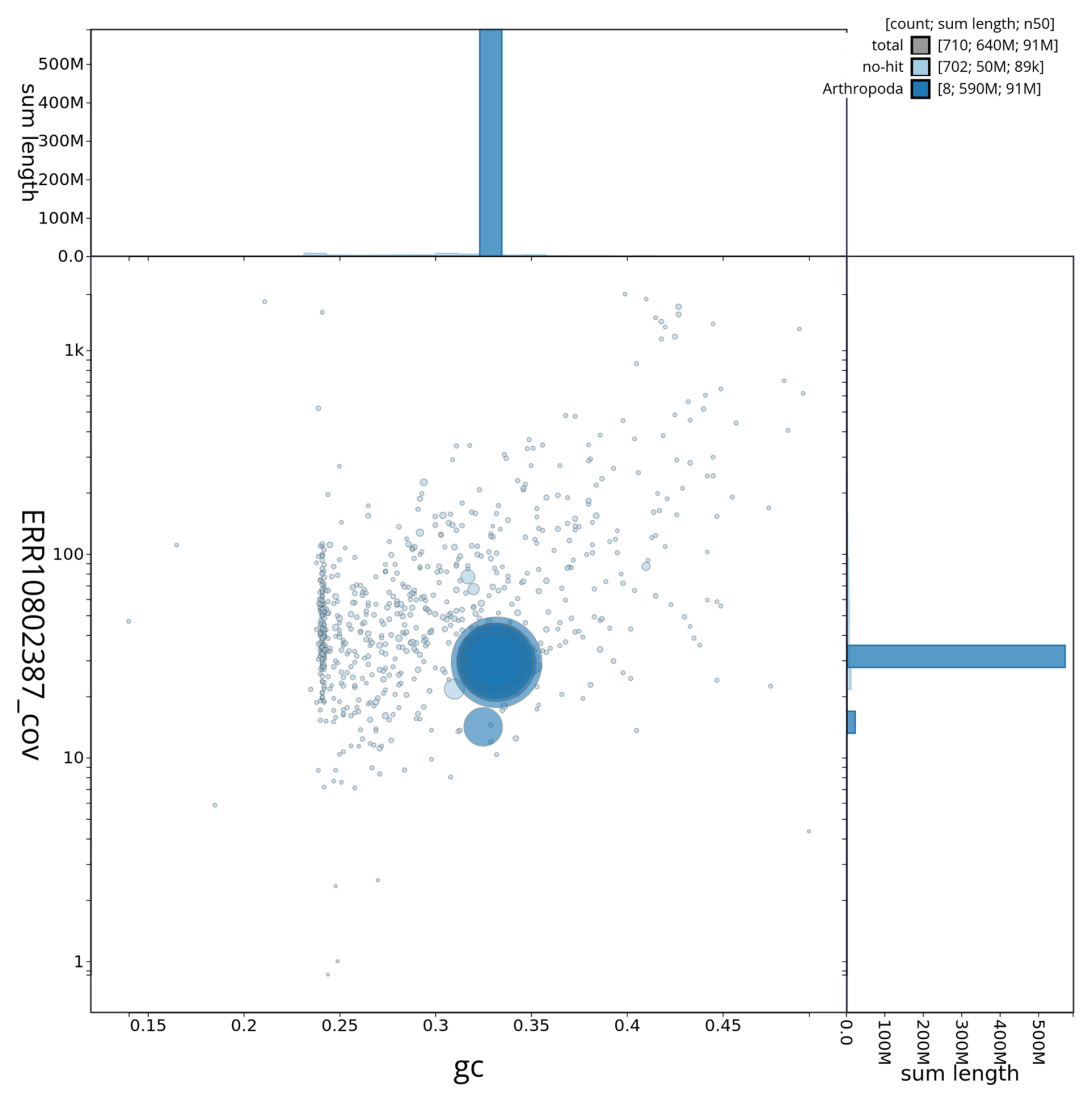
Genome assembly of *Crioceris asparagi*, icCriAspa1.1: BlobToolKit GC-coverage plot. BlobToolKit GC-coverage plot showing sequence coverage (vertical axis) and GC content (horizontal axis). The circles represent scaffolds, with the size proportional to scaffold length and the colour representing phylum membership. The histograms along the axes display the total length of sequences distributed across different levels of coverage and GC content. An interactive version of this figure is available at https://blobtoolkit.genomehubs.org/view/icCriAspa1_1/dataset/icCriAspa1_1/blob.

**Figure 4.**
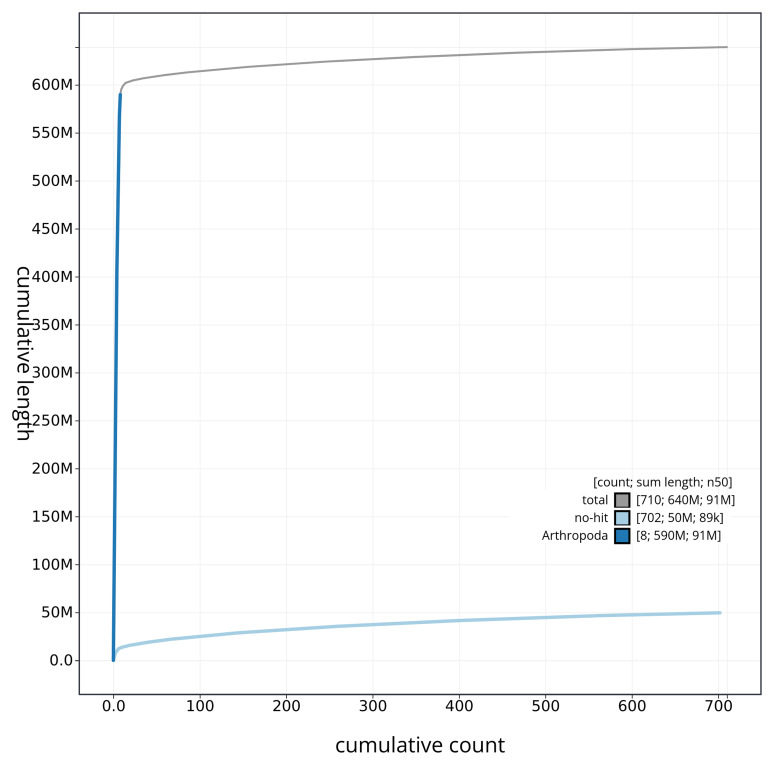
Genome assembly of *Crioceris asparagi* icCriAspa1.1: BlobToolKit cumulative sequence plot. The grey line shows cumulative length for all sequences. Coloured lines show cumulative lengths of sequences assigned to each phylum using the buscogenes taxrule. An interactive version of this figure is available at https://blobtoolkit.genomehubs.org/view/icCriAspa1_1/dataset/icCriAspa1_1/cumulative.

The snail plot in Figure 2 provides a summary of the assembly statistics, indicating the distribution of scaffold lengths and other assembly metrics. Figure 3 shows the distribution of scaffolds by GC proportion and coverage. Figure 4 presents a cumulative assembly plot, with separate curves representing different scaffold subsets assigned to various phyla, illustrating the completeness of the assembly.

**Figure 5.**
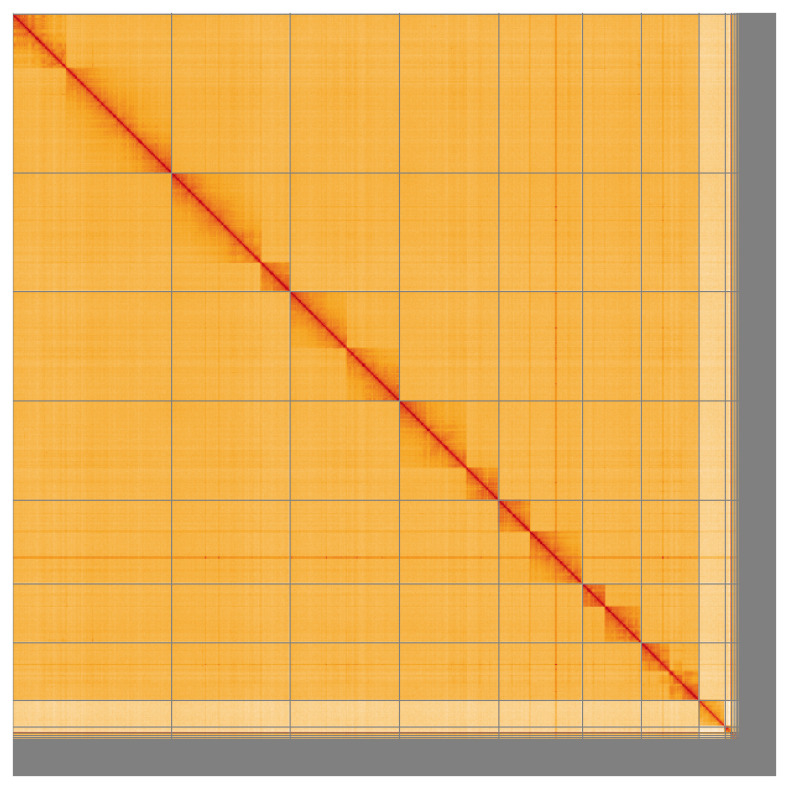
Genome assembly of *Crioceris asparagi* icCriAspa1.1: Hi-C contact map of the icCriAspa1.1 assembly, visualised using HiGlass. Chromosomes are shown in order of size from left to right and top to bottom. An interactive version of this figure may be viewed at https://genome-note-higlass.tol.sanger.ac.uk/l/?d=OtktpgA8SYaTECusDNb9xg.

Most of the assembly sequence (93.04%) was assigned to 9 chromosomal-level scaffolds, representing 7 autosomes and the X and Y sex chromosomes. These chromosome-level scaffolds, confirmed by the Hi-C data, are named in order of size ( Figure 5; Table 3). During manual curation it was noted that Chromosomes X and Y were assigned based on read coverage statistics. While not fully phased, the assembly deposited is of one haplotype. Contigs corresponding to the second haplotype have also been deposited. The mitochondrial genome was also assembled and can be found as a contig within the multifasta file of the genome submission, and as a separate fasta file with accession OY293835.1.

**Table 3.** Chromosomal pseudomolecules in the genome assembly of *Crioceris asparagi*, icCriAspa1.

INSDC accession	Name	Length (Mb)	GC%
OY293826.1	1	131.42	33.0
OY293827.1	2	97.98	33.0
OY293828.1	3	90.62	33.0
OY293829.1	4	82.3	33.0
OY293830.1	5	69.17	33.0
OY293831.1	6	48.75	33.0
OY293832.1	7	47.7	33.0
OY293833.1	X	21.83	32.5
OY293834.1	Y	5.05	31.0
OY293835.1	MT	0.02	24.0

The final assembly has a Quality Value (QV) of 58.8 and *k*-mer completeness of 98.64% for the combined assembly. BUSCO (v5.4.3) analysis using the endopterygota_odb10 reference set ( *n* = 2,124) indicated a completeness score of 98.4% (single = 97.6%, duplicated = 0.8%). The assembly achieves the EBP reference standard of 6.C.59. Other quality metrics are given in Table 2. 

## Genome annotation report

The *Crioceris asparagi* genome assembly (GCA_958507055.1) was annotated at the European Bioinformatics Institute (EBI) on Ensembl Rapid Release. The resulting annotation includes 26,898 transcribed mRNAs from 26,673 protein-coding genes ( Table 2; https://rapid.ensembl.org/Crioceris_asparagi_GCA_958507055.1/Info/Index). The average transcript length is 4,132.90, with an average of 3.55 exons per transcript.

## Methods

### Sample acquisition and DNA barcoding

An adult male specimen of *Crioceris asparagi* (specimen ID NHMUK014443406, ToLID icCriAspa1) was collected from Winsford Gardens, Penge, England, United Kingdom (latitude 51.41, longitude –0.06) on 2021-07-03. The specimen was collected and identified by Michael Geiser (Natural History Museum) and preserved by dry freezing at –80 °C.

The initial identification was verified by an additional DNA barcoding process according to the framework developed by Twyford *et al*. (2024). A small sample was dissected from the specimens and stored in ethanol, while the remaining parts were shipped on dry ice to the Wellcome Sanger Institute (WSI). The tissue was lysed, the COI marker region was amplified by PCR, and amplicons were sequenced and compared to the BOLD database, confirming the species identification ( Crowley *et al.*, 2023). Following whole genome sequence generation, the relevant DNA barcode region was also used alongside the initial barcoding data for sample tracking at the WSI ( Twyford *et al.*, 2024). The standard operating procedures for Darwin Tree of Life barcoding have been deposited on protocols.io ( Beasley *et al.*, 2023).

### Nucleic acid extraction

The workflow for high molecular weight (HMW) DNA extraction at the Wellcome Sanger Institute (WSI) Tree of Life Core Laboratory includes a sequence of procedures: sample preparation and homogenisation, DNA extraction, fragmentation and purification. Detailed protocols are available on protocols.io ( Denton *et al.*, 2023b).

The icCriAspa1 sample was prepared for DNA extraction by weighing and dissecting it on dry ice ( Jay *et al.*, 2023). Tissue from the abdomen was homogenised using a PowerMasher II tissue disruptor ( Denton *et al.*, 2023a).

HMW DNA was extracted in the WSI Scientific Operations core using the Automated MagAttract v2 protocol ( Oatley *et al.*, 2023). The DNA was sheared into an average fragment size of 12–20 kb in a Megaruptor 3 system ( Bates *et al.*, 2023). Sheared DNA was purified by solid-phase reversible immobilisation, using AMPure PB beads to eliminate shorter fragments and concentrate the DNA ( Strickland *et al.*, 2023). The concentration of the sheared and purified DNA was assessed using a Nanodrop spectrophotometer and Qubit Fluorometer using the Qubit dsDNA High Sensitivity Assay kit. Fragment size distribution was evaluated by running the sample on the FemtoPulse system.

### Hi-C preparation

Tissue from the head and thorax of the icCriAspa1 sample was processed at the WSI Scientific Operations core, using the Arima-HiC v2 kit. Tissue (stored at –80 °C) was fixed, and the DNA crosslinked using a TC buffer with 22% formaldehyde. After crosslinking, the tissue was homogenised using the Diagnocine Power Masher-II and BioMasher-II tubes and pestles. Following the kit manufacturer's instructions, crosslinked DNA was digested using a restriction enzyme master mix. The 5’-overhangs were then filled in and labelled with biotinylated nucleotides and proximally ligated. An overnight incubation was carried out for enzymes to digest remaining proteins and for crosslinks to reverse. A clean up was performed with SPRIselect beads prior to library preparation.

### Library preparation and sequencing

Library preparation and sequencing were performed at the WSI Scientific Operations core. Pacific Biosciences HiFi circular consensus DNA sequencing libraries were prepared using the PacBio Express Template Preparation Kit v2.0 (Pacific Biosciences, California, USA) as per the manufacturer's instructions. The kit includes the reagents required for removal of single-strand overhangs, DNA damage repair, end repair/A-tailing, adapter ligation, and nuclease treatment. Library preparation also included a library purification step using AMPure PB beads (Pacific Biosciences, California, USA) and size selection step to remove templates shorter than 3 kb using AMPure PB modified SPRI. DNA concentration was quantified using the Qubit Fluorometer v2.0 and Qubit HS Assay Kit and the final library fragment size analysis was carried out using the Agilent Femto Pulse Automated Pulsed Field CE Instrument and 165kb gDNA and 55kb BAC analysis kit. Samples were sequenced using the Sequel IIe system (Pacific Biosciences, California, USA). The concentration of the library loaded onto the Sequel IIe was in the range 40–135 pM. The SMRT link software, a PacBio web-based end-to-end workflow manager, was used to set-up and monitor the run, as well as perform primary and secondary analysis of the data upon completion.

For Hi-C library preparation, DNA was fragmented to a size of 400 to 600 bp using a Covaris E220 sonicator. The DNA was then enriched, barcoded, and amplified using the NEBNext Ultra II DNA Library Prep Kit following manufacturers’ instructions. The Hi-C sequencing was performed using paired-end sequencing with a read length of 150 bp on an Illumina NovaSeq 6000 instrument.

### Genome assembly, curation and evaluation

***Assembly***

The HiFi reads were first assembled using Hifiasm ( Cheng *et al.*, 2021) with the --primary option. Haplotypic duplications were identified and removed using purge_dups ( Guan *et al.*, 2020). The Hi-C reads were mapped to the primary contigs using bwa-mem2 ( Vasimuddin *et al.*, 2019). The contigs were further scaffolded using the provided Hi-C data ( Rao *et al.*, 2014) in YaHS ( Zhou *et al.*, 2023) using the --break option for handling potential misassemblies. The scaffolded assemblies were evaluated using Gfastats ( Formenti *et al.*, 2022), BUSCO ( Manni *et al.*, 2021) and MERQURY.FK ( Rhie *et al.*, 2020).

The mitochondrial genome was assembled using MitoHiFi ( Uliano-Silva *et al.*, 2023), which runs MitoFinder ( Allio *et al.*, 2020) and uses these annotations to select the final mitochondrial contig and to ensure the general quality of the sequence.

***Assembly curation***

The assembly was decontaminated using the Assembly Screen for Cobionts and Contaminants (ASCC) pipeline (article in preparation). Flat files and maps used in curation were generated in TreeVal ( Pointon *et al.*, 2023). Manual curation was primarily conducted using PretextView ( Harry, 2022), with additional insights provided by JBrowse2 ( Diesh *et al.*, 2023) and HiGlass ( Kerpedjiev *et al.*, 2018). Scaffolds were visually inspected and corrected as described by Howe *et al*. (2021). Any identified contamination, missed joins, and mis-joins were corrected, and duplicate sequences were tagged and removed. The curation process is documented at https://gitlab.com/wtsi-grit/rapid-curation (article in preparation).

***Evaluation of the final assembly***

A Hi-C map for the final assembly was produced using bwa-mem2 ( Vasimuddin *et al.*, 2019) in the Cooler file format ( Abdennur & Mirny, 2020). To assess the assembly metrics, the *k*-mer completeness and QV consensus quality values were calculated in Merqury ( Rhie *et al.*, 2020). This work was done using Nextflow ( Di Tommaso *et al.*, 2017) DSL2 pipelines “sanger-tol/readmapping” ( Surana *et al.*, 2023a) and “sanger-tol/genomenote” ( Surana *et al.*, 2023b). The genome was analysed within the BlobToolKit environment ( Challis *et al.*, 2020) and BUSCO scores ( Manni *et al.*, 2021) were calculated.

The genome assembly and evaluation pipelines were developed using nf-core tooling ( Ewels *et al.*, 2020) and MultiQC ( Ewels *et al.*, 2016), relying on the Conda package manager, the Bioconda initiative ( Grüning *et al.*, 2018), the Biocontainers infrastructure ( da Veiga Leprevost *et al.*, 2017), as well as the Docker ( Merkel, 2014) and Singularity ( Kurtzer *et al.*, 2017) containerisation solutions.

Table 4 contains a list of relevant software tool versions and sources.

**Table 4.** Software tools: versions and sources.

Software tool	Version	Source
BlobToolKit	4.2.1	https://github.com/blobtoolkit/blobtoolkit
BUSCO	5.3.2	https://gitlab.com/ezlab/busco
bwa-mem2	2.2.1	https://github.com/bwa-mem2/bwa-mem2
Cooler	0.8.11	https://github.com/open2c/cooler
FastK	427104ea91c78c3b8b8b49f1a7d6bbeaa869ba1c	https://github.com/thegenemyers/FASTK
Gfastats	1.3.6	https://github.com/vgl-hub/gfastats
Hifiasm	0.16.1-r375	https://github.com/chhylp123/hifiasm
HiGlass	44086069ee7d4d3f6f3f0012569789ec138f42b84 aa44357826c0b6753eb28de	https://github.com/higlass/higlass
Merqury.FK	d00d98157618f4e8d1a9190026b19b471055b2 2e	https://github.com/thegenemyers/MERQURY.FK
MitoHiFi	2	https://github.com/marcelauliano/MitoHiFi
Nextflow	23.04.0-5857	https://github.com/nextflow-io/nextflow
PretextView	0.2	https://github.com/sanger-tol/PretextView
purge_dups	1.2.3	https://github.com/dfguan/purge_dups
sanger-tol/ascc	-	https://github.com/sanger-tol/ascc
sanger-tol/genomenote	v1.0	https://github.com/sanger-tol/genomenote
sanger-tol/readmapping	1.1.0	https://github.com/sanger-tol/readmapping
Singularity	3.9.0	https://github.com/sylabs/singularity
YaHS	1.2a	https://github.com/c-zhou/yahs

### Genome annotation

The BRAKER2 pipeline ( Brůna *et al.*, 2021) was used in the default protein mode to generate annotation for the *Crioceris asparagi* assembly (GCA_958507055.1) in Ensembl Rapid Release at the EBI.

### Wellcome Sanger Institute – Legal and Governance

The materials that have contributed to this genome note have been supplied by a Darwin Tree of Life Partner. The submission of materials by a Darwin Tree of Life Partner is subject to the **‘Darwin Tree of Life Project Sampling Code of Practice’**, which can be found in full on the Darwin Tree of Life website here. By agreeing with and signing up to the Sampling Code of Practice, the Darwin Tree of Life Partner agrees they will meet the legal and ethical requirements and standards set out within this document in respect of all samples acquired for, and supplied to, the Darwin Tree of Life Project. 

Further, the Wellcome Sanger Institute employs a process whereby due diligence is carried out proportionate to the nature of the materials themselves, and the circumstances under which they have been/are to be collected and provided for use. The purpose of this is to address and mitigate any potential legal and/or ethical implications of receipt and use of the materials as part of the research project, and to ensure that in doing so we align with best practice wherever possible. The overarching areas of consideration are:

• Ethical review of provenance and sourcing of the material

• Legality of collection, transfer and use (national and international)

Each transfer of samples is further undertaken according to a Research Collaboration Agreement or Material Transfer Agreement entered into by the Darwin Tree of Life Partner, Genome Research Limited (operating as the Wellcome Sanger Institute), and in some circumstances other Darwin Tree of Life collaborators.

## Data Availability

European Nucleotide Archive: Crioceris asparagi (common asparagus beetle). Accession number PRJEB59077; https://identifiers.org/ena.embl/PRJEB59077. The genome sequence is released openly for reuse. The *Crioceris asparagi* genome sequencing initiative is part of the Darwin Tree of Life (DToL) project. All raw sequence data and the assembly have been deposited in INSDC databases. Raw data and assembly accession identifiers are reported in Table 1 and Table 2. Metadata for specimens, BOLD barcode results, spectra estimates, sequencing runs, contaminants and pre-curation assembly statistics are given at https://links.tol.sanger.ac.uk/species/131627.

## Author information

Members of the Natural History Museum Genome Acquisition Lab are listed here: https://doi.org/10.5281/zenodo.12159242.

Members of the Darwin Tree of Life Barcoding collective are listed here: https://doi.org/10.5281/zenodo.12158331.

Members of the Wellcome Sanger Institute Tree of Life Management, Samples and Laboratory team are listed here: https://doi.org/10.5281/zenodo.12162482.

Members of Wellcome Sanger Institute Scientific Operations: Sequencing Operations are listed here: https://doi.org/10.5281/zenodo.12165051.

Members of the Wellcome Sanger Institute Tree of Life Core Informatics team are listed here: https://doi.org/10.5281/zenodo.12160324.

Members of the Tree of Life Core Informatics collective are listed here: https://doi.org/10.5281/zenodo.12205391.

Members of the Darwin Tree of Life Consortium are listed here: https://doi.org/10.5281/zenodo.4783558.

## References

[ref-1] Abdennur N Mirny LA : Cooler: scalable storage for Hi-C data and other genomically labeled arrays. *Bioinformatics.* 2020;36(1):311–316. 10.1093/bioinformatics/btz540 31290943 PMC8205516

[ref-2] Allio R Schomaker-Bastos A Romiguier J : MitoFinder: efficient automated large-scale extraction of mitogenomic data in target enrichment phylogenomics. *Mol Ecol Resour.* 2020;20(4):892–905. 10.1111/1755-0998.13160 32243090 PMC7497042

[ref-5] Bates A Clayton-Lucey I Howard C : Sanger Tree of Life HMW DNA fragmentation: diagenode Megaruptor ^®^3 for LI PacBio. *protocols.io.* 2023. 10.17504/protocols.io.81wgbxzq3lpk/v1

[ref-6] Beasley J Uhl R Forrest LL : DNA barcoding SOPs for the Darwin Tree of Life project. *protocols.io.* 2023; [Accessed 25 June 2024]. 10.17504/protocols.io.261ged91jv47/v1

[ref-60] Bezděk J Sekerka L : Catalogue of palaearctic coleoptera. Volume 6/2/1. Updated and revised second edition. Chrysomeloidea II (Orsodacnidae, Megalopodidae, Chrysomelidae).Leiden: Brill,2024. 10.1163/9789004443303

[ref-3] Brůna T Hoff KJ Lomsadze A : BRAKER2: Automatic eukaryotic genome annotation with GeneMark-EP+ and AUGUSTUS supported by a protein database. *NAR Genom Bioinform.* 2021;3(1): lqaa108. 10.1093/nargab/lqaa108 33575650 PMC7787252

[ref-10] Challis R Richards E Rajan J : BlobToolKit – interactive quality assessment of genome assemblies. *G3 (Bethesda).* 2020;10(4):1361–1374. 10.1534/g3.119.400908 32071071 PMC7144090

[ref-11] Cheng H Concepcion GT Feng X : Haplotype-resolved *de novo* assembly using phased assembly graphs with hifiasm. *Nat Methods.* 2021;18(2):170–175. 10.1038/s41592-020-01056-5 33526886 PMC7961889

[ref-61] Chittenden FH : The asparagus beetles and their control.Washington, DC: United States Department of Agriculture,1917. Reference Source

[ref-62] Cox ML : Atlas of the Seed and Leaf Beetles of Britain.Newbury: Pisces Publications,2007. Reference Source

[ref-12] Crowley L Allen H Barnes I : A sampling strategy for genome sequencing the British terrestrial arthropod fauna [version 1; peer review: 2 approved]. *Wellcome Open Res.* 2023;8:123. 10.12688/wellcomeopenres.18925.1 37408610 PMC10318377

[ref-13] da Veiga Leprevost F Grüning BA Alves Aflitos S : BioContainers: an open-source and community-driven framework for software standardization. *Bioinformatics.* 2017;33(16):2580–2582. 10.1093/bioinformatics/btx192 28379341 PMC5870671

[ref-15] Denton A Oatley G Cornwell C : Sanger Tree of Life sample homogenisation: PowerMash. *protocols.io.* 2023a. 10.17504/protocols.io.5qpvo3r19v4o/v1

[ref-16] Denton A Yatsenko H Jay J : Sanger Tree of Life wet laboratory protocol collection V.1. *protocols.io.* 2023b. 10.17504/protocols.io.8epv5xxy6g1b/v1

[ref-17] Di Tommaso P Chatzou M Floden EW : Nextflow enables reproducible computational workflows. *Nat Biotechnol.* 2017;35(4):316–319. 10.1038/nbt.3820 28398311

[ref-18] Diesh C Stevens GJ Xie P : JBrowse 2: a modular genome browser with views of synteny and structural variation. *Genome Biol.* 2023;24(1): 74. 10.1186/s13059-023-02914-z 37069644 PMC10108523

[ref-63] Duff AG : Beetles of Britain and Ireland. Vol. 4: Cerambycidae to Curculionidae.West Runton: A.G. Duff publishing,2016. Reference Source

[ref-64] Duff AG : Checklist of Beetles of the British Isles, 3rd Edition.Iver: Pemberley Books,2018. Reference Source

[ref-20] Ewels P Magnusson M Lundin S : MultiQC: summarize analysis results for multiple tools and samples in a single report. *Bioinformatics.* 2016;32(19):3047–3048. 10.1093/bioinformatics/btw354 27312411 PMC5039924

[ref-19] Ewels PA Peltzer A Fillinger S : The nf-core framework for community-curated bioinformatics pipelines. *Nat Biotechnol.* 2020;38(3):276–278. 10.1038/s41587-020-0439-x 32055031

[ref-21] Formenti G Abueg L Brajuka A : Gfastats: conversion, evaluation and manipulation of genome sequences using assembly graphs. *Bioinformatics.* 2022;38(17):4214–4216. 10.1093/bioinformatics/btac460 35799367 PMC9438950

[ref-65] Fowler WW : The coleoptera of the British Islands, Lamellicornia - Serricornia - Longicornia - Phytophaga.London: L. Reeve & Co,1890.

[ref-22] Grüning B Dale R Sjödin A : Bioconda: sustainable and comprehensive software distribution for the life sciences. *Nat Methods.* 2018;15(7):475–476. 10.1038/s41592-018-0046-7 29967506 PMC11070151

[ref-23] Guan D McCarthy SA Wood J : Identifying and removing haplotypic duplication in primary genome assemblies. *Bioinformatics.* 2020;36(9):2896–2898. 10.1093/bioinformatics/btaa025 31971576 PMC7203741

[ref-24] Harry E : PretextView (Paired REad TEXTure Viewer): a desktop application for viewing pretext contact maps. 2022. Reference Source

[ref-66] Heinze E : Die criocerinenAfrikas (Col. Chrysomelidae). *EntomologischeArbeiten Aus Dem Museum G. Frey.* 1962;13:156–270.

[ref-25] Howe K Chow W Collins J : Significantly improving the quality of genome assemblies through curation. *GigaScience.* 2021;10(1): giaa153. 10.1093/gigascience/giaa153 33420778 PMC7794651

[ref-26] Jay J Yatsenko H Narváez-Gómez JP : Sanger Tree of Life sample preparation: triage and dissection. *protocols.io.* 2023. 10.17504/protocols.io.x54v9prmqg3e/v1

[ref-27] Kerpedjiev P Abdennur N Lekschas F : HiGlass: web-based visual exploration and analysis of genome interaction maps. *Genome Biol.* 2018;19(1): 125. 10.1186/s13059-018-1486-1 30143029 PMC6109259

[ref-67] Kimoto S Gressitt JL : Chrysomelidae (Coleoptera) of Thailand, Cambodia, Laos and Vietnam. I. Sagrinae, Donaciinae, Zeugophorinae, Megalopodinae and Criocerinae. *Pacific Insects.* 1979;20(2–3):191–256. Reference Source

[ref-28] Kurtzer GM Sochat V Bauer MW : Singularity: scientific containers for mobility of compute. *PLoS One.* 2017;12(5): e0177459. 10.1371/journal.pone.0177459 28494014 PMC5426675

[ref-30] Manni M Berkeley MR Seppey M : BUSCO update: novel and streamlined workflows along with broader and deeper phylogenetic coverage for scoring of eukaryotic, prokaryotic, and viral genomes. *Mol Biol Evol.* 2021;38(10):4647–4654. 10.1093/molbev/msab199 34320186 PMC8476166

[ref-31] Merkel D : Docker: lightweight Linux containers for consistent development and deployment. *Linux J.* 2014;2014(239): 2, [Accessed 2 April 2024]. Reference Source

[ref-68] Monrós F : Los géneros de chrysomelidae (Coleoptera). *Opera Lilloana.* 3[1959],1960;5–337, pls. 1–3. Reference Source

[ref-69] NBN Atlas Partnership: *Crioceris asparagi* (Linnaeus, 1758).Asparagus Beetle map on the NBN Atlas,2024. Reference Source

[ref-33] Oatley G Denton A Howard C : Sanger Tree of Life HMW DNA extraction: automated MagAttract v.2. *protocols.io.* 2023. 10.17504/protocols.io.kxygx3y4dg8j/v1

[ref-51] Pointon DL Eagles W Sims Y : sanger-tol/treeval v1.0.0 – Ancient Atlantis. 2023. 10.5281/zenodo.10047653

[ref-37] Rao SSP Huntley MH Durand NC : A 3D map of the human genome at kilobase resolution reveals principles of chromatin looping. *Cell.* 2014;159(7):1665–1680. 10.1016/j.cell.2014.11.021 25497547 PMC5635824

[ref-70] Rheinheimer J Hassler M : Die blattkäfer baden-Württembergs.Karlsruhe: Kleinsteuber Books,2018. Reference Source

[ref-38] Rhie A McCarthy SA Fedrigo O : Towards complete and error-free genome assemblies of all vertebrate species. *Nature.* 2021;592(7856):737–746. 10.1038/s41586-021-03451-0 33911273 PMC8081667

[ref-39] Rhie A Walenz BP Koren S : Merqury: reference-free quality, completeness, and phasing assessment for genome assemblies. *Genome Biol.* 2020;21(1): 245. 10.1186/s13059-020-02134-9 32928274 PMC7488777

[ref-71] Steinhausen W : 116. Familie: Chrysomelidae.In: Klausnitzer, B. (ed.) *Die KäferMitteleuropas. Larven 2: Myxophaga, Polyphaga Teil 1.*Krefeld: Goecke & Evers,1994;231–314.

[ref-72] Steinhausen W : Die puppenmitteleuropäischerBlattkäfer - Eine vorläufigeBestimmungstabelle. 1. Teil (Coleoptera: Chrysomelidae). *Mitt Munch Entomol Ges.* 2001;91:35–63. Reference Source

[ref-36] Strickland M Cornwell C Howard C : Sanger Tree of Life fragmented DNA clean up: manual SPRI. *protocols.io.* 2023. 10.17504/protocols.io.kxygx3y1dg8j/v1

[ref-40] Surana P Muffato M Qi G : Sanger-tol/readmapping: sanger-tol/readmapping v1.1.0 - Hebridean Black (1.1.0). *Zenodo.* 2023a. 10.5281/zenodo.7755669

[ref-41] Surana P Muffato M Sadasivan Baby C : sanger-tol/genomenote (v1.0.dev). *Zenodo.* 2023b. 10.5281/zenodo.6785935

[ref-42] Twyford AD Beasley J Barnes I : A DNA barcoding framework for taxonomic verification in the darwin Tree of Life project [version 1; peer review: 2 approved]. *Wellcome Open Res.* 2024;9:339. 10.12688/wellcomeopenres.21143.1 39386966 PMC11462125

[ref-48] Uliano-Silva M Ferreira JGRN Krasheninnikova K : MitoHiFi: a python pipeline for mitochondrial genome assembly from PacBio high fidelity reads. *BMC Bioinformatics.* 2023;24(1): 288. 10.1186/s12859-023-05385-y 37464285 PMC10354987

[ref-49] Vasimuddin M Misra S Li H : Efficient architecture-aware acceleration of BWA-MEM for multicore systems.In: *2019 IEEE International Parallel and Distributed Processing Symposium (IPDPS).*IEEE,2019;314–324. 10.1109/IPDPS.2019.00041

[ref-73] Vencl FV Leschen RAB : 2.7.6. Criocerinae Latreille, 1807,In: Leschen, R. A. B. and Beutel, R. G. (eds.) *Handbook of Zoology Arthropoda: Insecta: Coleoptera: Volume 3: Morphology and Systematics (Phytophaga).*Berlin: De Gruyter,2014;237–242. Reference Source

[ref-74] Warchałowski A : The palaearctic chrysomelidae. Identification keys. Vols. 1 & 2.Warszawa: Natura Optima Dux Foundation,2010.

[ref-50] Zhou C McCarthy SA Durbin R : YaHS: yet another Hi-C scaffolding tool. *Bioinformatics.* 2023;39(1): btac808. 10.1093/bioinformatics/btac808 36525368 PMC9848053

