# Supplementary material for: The genome sequence of the Asparagus Beetle, Crioceris asparagi (Linnaeus, 1758)
Source: Wellcome Open Res. 2025 Jan 15;10:18. [Version 1] doi: 10.12688/wellcomeopenres.23460.1 (PMC12171721; doi:10.12688/wellcomeopenres.23460.1)
Supplement: Peer review history

# Reviewer report: Reviewer response for version 1

**DOI:** 10.21956/wellcomeopenres.25870.r125376

**Published:** 2025-08-23

**Authors:** Terrence Sylvester

**Affiliations:** 1 University of Memphis, Tennessee, USA

The author presents the genome assembly of the Asparagus Beetle, *Crioceris asparagi*. The authors start by providing a brief but adequate amount of information on the species’ distribution, life history, and morphology. The current context is sufficient to support the value of a high-quality reference genome for this species.

 Based on standard genome assembly quality metrics, the genome is of excellent quality. The authors also report the tissue location they used to generate each sequencing type, which I think is excellent for repeatability. 

 Other than that, I do not have any significant remarks.

Are sufficient details of methods and materials provided to allow replication by others?

Yes

Is the rationale for creating the dataset(s) clearly described?

Yes

Are the datasets clearly presented in a useable and accessible format?

Yes

Are the protocols appropriate and is the work technically sound?

Yes

Reviewer Expertise:

Genomics

I confirm that I have read this submission and believe that I have an appropriate level of expertise to confirm that it is of an acceptable scientific standard.

# Reviewer report: Reviewer response for version 1

**DOI:** 10.21956/wellcomeopenres.25870.r125374

**Published:** 2025-06-16

**Authors:** Chenyang Cai

**Affiliations:** 1 Chinese Academy of Sciences, Nanjing, China

The manuscript presents a high-quality genome assembly of *Crioceris asparagi,* providing valuable genomic resources for this economically significant pest. The assembly is well-structured, with 93.04% of the sequence scaffolded into nine chromosomal pseudomolecules, including the X and Y sex chromosomes—an impressive achievement given the challenges of beetle genome assembly. The inclusion of the mitochondrial genome (15.76 kb) further enhances the dataset's utility for evolutionary and phylogenetic studies. The annotation of 26,673 protein-coding genes via Ensembl is robust and aligns with expectations for Coleoptera, though additional functional validation could further strengthen gene predictions.

 In formal academic writing, the genus name should be written in full when first mentioned in a text, and may be abbreviated in subsequent references if the context is clear. Based on your request, the corrected sentence should read:

 ​​" *Crioceris* *asparagi* cannot easily be..."​​

Are sufficient details of methods and materials provided to allow replication by others?

Yes

Is the rationale for creating the dataset(s) clearly described?

Yes

Are the datasets clearly presented in a useable and accessible format?

Yes

Are the protocols appropriate and is the work technically sound?

Yes

Reviewer Expertise:

beetles; phylogenomics

I confirm that I have read this submission and believe that I have an appropriate level of expertise to confirm that it is of an acceptable scientific standard.

# Reviewer report: Reviewer response for version 1

**DOI:** 10.21956/wellcomeopenres.25870.r117626

**Published:** 2025-01-29

**Authors:** Doga Cedden

**Affiliations:** 1 University of Göttingen, Göttingen, Germany

Geiser et al. report a high-quality genome assembly from *Crioceris asparagi. *The background information is sufficient, and Fig. 1 provides a specimen photograph. The authors provide a sufficient quality assessment of their assembly. The quality of the assembly is adequate, albeit the *k*-mer completeness is a bit lower than other submissions from the Darwin Tree of Life Consortium (usually 100%). The data presented in the data note is publicly accessible.

 I suggest providing a scale in the photographs provided in Fig. 1.

Are sufficient details of methods and materials provided to allow replication by others?

Yes

Is the rationale for creating the dataset(s) clearly described?

Yes

Are the datasets clearly presented in a useable and accessible format?

Yes

Are the protocols appropriate and is the work technically sound?

Yes

Reviewer Expertise:

I work on coleopteran pests using molecular methods, including RNAi and RNA-seq.

I confirm that I have read this submission and believe that I have an appropriate level of expertise to confirm that it is of an acceptable scientific standard.
